# The Rice miR396-GRF-GIF-SWI/SNF Module: A Player in GA Signaling

**DOI:** 10.3389/fpls.2021.786641

**Published:** 2022-01-11

**Authors:** Yuzhu Lu, Jia Zeng, Qiaoquan Liu

**Affiliations:** ^1^College of Bioscience and Biotechnology, Yangzhou University, Yangzhou, China; ^2^Joint International Research Laboratory of Agriculture and Agri-Product Safety, The Ministry of Education of China, Yangzhou University, Yangzhou, China; ^3^Key Laboratory of Plant Functional Genomics of the Ministry of Education, College of Agriculture, Yangzhou University, Yangzhou, China

**Keywords:** GRF, GIF, SWI/SNF, DELLA, GA signaling

## Abstract

Rice *Growth-Regulating Factors* (*GRFs*) were originally identified to be gibberellin (GA)-induced, but the nature of GA induction has remained unknown because most reports thereafter focused on revealing their roles in growth-promoting activities. GRFs have the WRC (Trp, Arg, Cys) domain to target DNA and contain the QLQ (Gln, Leu, Gln) domain to interact with GRF-Interacting Factor (GIF), which recruits ATP-dependent DNA translocase Switch/Sucrose Non-fermenting (SWI/SNF) for chromatin remodeling. Both GRFs and GIFs exhibit transcriptional activities but GIFs lack a DNA-binding domain. So, GRFs act like a navigator in the GRF-GIF-SWI/SNF complex, determining when and where the complex should work on. The levels of most rice *GRFs* can be sensitively regulated by miR396, which responds to many developmental and environmental factors. Recent clues from several studies highlight the original question of how GRFs participate in GA signaling. DELLA (contain DELLA motif) protein plays dual roles in controlling the level of GRFs by regulating the level of miR396 and interacting with GRFs. Here we address the question of why this complex plays an essential role in controlling plant growth focusing on the action of GA signaling pivot, DELLA.

## Introduction

Two decades ago, the first member of the *Growth-Regulating Factor* (*GRF)* family, *OsGRF1*, was identified as a gibberellin (GA)-induced gene in the internodes of rice (van der Knaap and Kende, 1995; van der Knaap et al., 2000). Subsequently, 12 *GRF* members were found in rice and most of them were proved to be GA-induced (Choi et al., 2004). The *GRF* family has been identified experimentally or computationally in many plant species and is deemed as a class of deeply conserved transcription factors in land plants (Omidbakhshfard et al., 2015; Kim, 2019). So far, growing evidence has shown that *GRFs* promote the growth of various plant organs, such as leaf expansion, stem and root elongation, seed and flower formation, etc. Very few were reported to inhibit growth (Omidbakhshfard et al., 2015; Kim, 2019). Since the last comprehensive review articles mainly focused on collecting and summarizing the individual roles of this family, here we present a new landscape of how this family and partners work together to take part in GA signaling. Our updated outline could provide new insights into a better understanding of the functions of the miR396-GRF-GIF-SWI/SNF module.

## GRFs: Navigator in the Positive Complex of GRF- GRF-Interacting Factor (GIF)- Switch/Sucrose Non-Fermenting (SWI/SNF)

The roles of GRFs as transcription factors may be attributed to their WRC (Trp, Arg, Cys) domains in the N-terminus which have DNA-binding abilities as well as their transcriptional activities in C-terminus (van der Knaap et al., 2000; Kim et al., 2003; Choi et al., 2004). Another characteristic domain in GRFs is the QLQ (Gln, Leu, Gln) motif which is used for interacting with GIFs (Kim and Kende, 2004; Horiguchi et al., 2005). GRFs prompt growth *via* working with GIFs, which recruit the ATP-dependent DNA translocase SWI/SNF for chromatin remodeling (Vercruyssen et al., 2014). *GIF* genes are seemed to be more deeply conserved than *GRF* genes. *GIFs* exist in most eukaryotic organisms, such as embryophytes, “green algae,” and metazoans, implying probably more molecular roles (Kim and Kende, 2004; de Bruijn and Geurts van Kessel, 2006). Furthermore, unlike *GRFs* which contain 8–20 members in “higher” plant genomes, *GIFs* usually comprise lower copies of about 2–5 members in plant species (Omidbakhshfard et al., 2015). Besides interacting with GRFs, GIF may also work with other factors in promoting plant growth (Debernardi et al., 2014; Vercruyssen et al., 2014; Nelissen et al., 2015; Zhang et al., 2018). GIF has been also proved to have transcriptional activities, but it lacks a DNA-binding domain, suggesting its role in transcription may be a co-regulator (Vercruyssen et al., 2014; Zhang et al., 2018).

**Figure 1:**
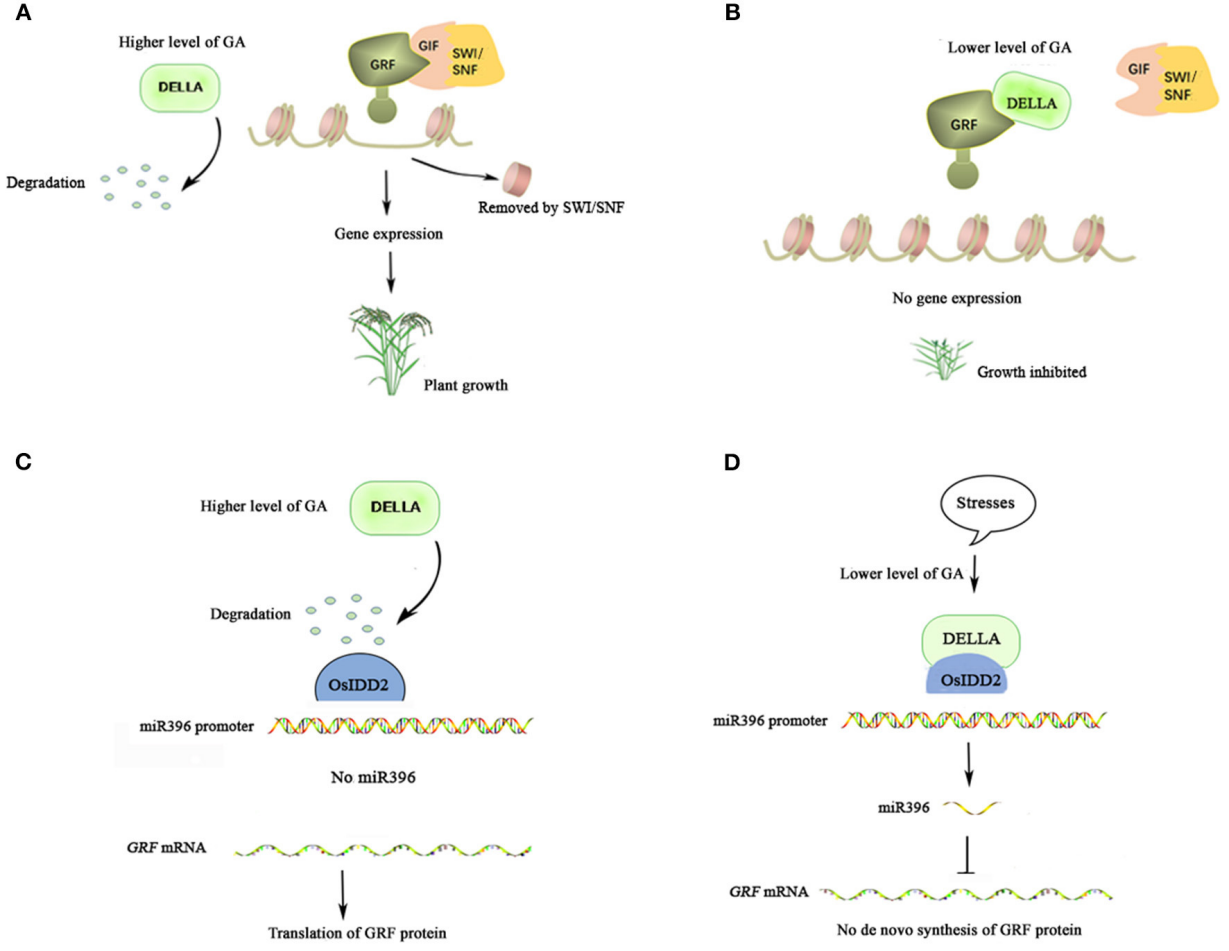
Rice miR396-growth regulating factors (GRF)-GRF Interacting factor (GIF)- Switch/Sucrose Non-fermenting SWI/SNF module in gibrellin (GA) signaling. **(A)** GRF-GIF-SWI/SNF module promotes plant growth. DELLA is degraded in the presence of a higher level of GA at meristematic organs or under suitable conditions, then GRF interacts with GIF which recruits SWI/SNF to form a positive complex for promoting plant growth. **(B)** DELLA antagonizes GRF under adverse conditions. A higher level of DELLA mediated by a lower level of GA under adverse conditions interacts with GRF. The separation between GRF and GIF mediated by DELLA leads to inhibition of plant growth. **(C)** *GRFs* are induced by GA. At meristematic organs or under suitable conditions along with a higher level of GA, degraded DELLA leads to a lower miR396 level which in turn results in a higher level of *GRFs* mRNA for translating protein. **(D)** miR396 suppresses *GRFs* mRNA under stress. A higher level of DELLA mediated by lower GA under stresses interacts with OsIDD2 to promote the expression of miR396 which in turn knocks down *GRFs* mRNA.

The functions of some identified targets of GRFs seemed to be diverse, for instance, *DEHYDRATION-RESPONSIVE ELEMENT BINDING PROTEIN2A* (*DREB2A*) of AtGRF7 (Kim et al., 2012) and *OBP3-RESPONSIVE GENE3* (*ORG3*) of AtGRF9 (Omidbakhshfard et al., 2018) in *Arabidopsis*. However, numerous clues indicated that GRFs execute their functions mainly through prompting cell proliferation in a broad range of organs (Ercoli et al., 2016). The time course of gene expression and analysis of cell division activity in the intercalary meristem suggest that *OsGRF1* may mediate an early event in the cell cycle (van der Knaap et al., 2000). Nowadays, with a deepening understanding, it has been known that the positive roles of GRFs in regulating growth also require the partners of GIFs and SWI/SNF. To facilitate gene expressions that are responsible for the transition from stem cells to rapidly dividing daughter cells, the transcriptional machinery depends on chromatin remodeling complexes, such as SWI/SNF, to loosen the association between DNA and histone octamers (Figure 1A) (Clapier and Cairns, 2009). In this regard, the GRF-GIF-SWI/SNF module acts as a growth-promoting complex, in which GRF serves as a navigator determining, through its WRC domain, which places in the genome is supposed to be targeted. In rice and *Arabidopsis*, besides some common tissues such as shoot apical meristem (SAM) where almost all members of this family prefer to accumulate (Kim et al., 2003; Choi et al., 2004), the expression patterns of different GRFs overall tend to display a space- or time-specific manner (van der Knaap et al., 2000; Choi et al., 2004; Lu et al., 2020). The accumulation of a given GRF member in a specific tissue usually defines its growth-promoting roles in that organ.

## Della: Antagonizing GRFs Under Adverse Conditions

Even though GRFs are deemed as positive factors in promoting growth, surprisingly, they have been recently found to be able to interact with a negative regulator of GA signaling, DELLA (Li et al., 2018; Lantzouni et al., 2020). DELLA proteins act as growth repressors by inhibiting GA signaling in response to many conditions. GA, as one kind of classic plant hormone, regulates key processes of plant development and determines many traits of agronomic performances (Davière and Achard, 2013). Similar to GIFs, DELLA also lacks a DNA-binding domain but acts as transcriptional co-regulators (Ito et al., 2018). A number of studies demonstrated that DELLA proteins can interact with a list of DELLA-interacting proteins (DIPs) *via* transactivation or sequestration (Van De Velde et al., 2017). “Transactivation” means that DELLA can work with some transcription factors to promote the expression of the related genes, usually of growth repressors, while “sequestration” means that DELLA can prevent transcript factors from promoting the expression of the related genes, usually of growth activators (Van De Velde et al., 2017). Interestingly, several recent studies showed that DELLA can control GRFs *via* these two means (Li et al., 2018; Lantzouni et al., 2020; Lu et al., 2020).

OsGRF4 was discovered to improve agricultural performances by increasing grain size and yield (Che et al., 2015; Duan et al., 2015; Li et al., 2016), which could be ascribed to its ability to prompt inorganic nitrogen uptake and carbon fixation (Li et al., 2018). This contrasts with the property of DELLA, which inhibits them. Further evidence demonstrated that DELLA can interact with OsGRF4 and competitively inhibits the OsGRF4-OsGIF1 interaction. Interestingly, OsGRF4 abundance is self-promoted and DELLA inhibits that promotion even though the mechanism is unclear (Li et al., 2018). Furthermore, extensive interactions between OsSLR1 and OsGRFs were observed, although their physiologic meanings were not specified (Li et al., 2018). It is well-known that higher and stable levels of DELLAs exist in plants in the absence of GA (Ito et al., 2018). So, under lower GA concentrations, the interactions between abundant DELLAs, and GRFs would limit the growth-promoting roles of the GRF-GIF complex (Figure 1B). This view has been further supported by a study that shows that the extensive interactions of DELLAs and ATGRFs exist in response to cold stress (Lantzouni et al., 2020). In the meantime, the expression levels of some GA biosynthesis genes and endogenous levels of the bioactive GAs are decreased under low temperatures (Sakata et al., 2014). In fact, besides low temperature, various stresses, including salt and osmotic stress, can also reduce GA levels, which in turn increase the level of DELLA proteins (Colebrook et al., 2014). Given that, the interactions between DELLAs and GRFs facilitated by reduced levels of GAs under various stresses would result in growth inhibition and thus, probably be a strategy for plants to survive in adverse conditions.

## Della-Controlled miR396: A Sensitive Regulatory Switch of GRF Complex

The interactions of SLR1 and OsGRFs at the protein level still cannot fully address the original question of why the expression of *OsGRFs* mRNA occurs in a low level under a low level of GA. So it is possible there might be another regulatory way that controls the expression of *GRFs* under the influence of GA. miR396 is an evolutionarily conserved miRNA that negatively regulates the abundance of *GRFs* mRNA (Jones-Rhoades and Bartel, 2004; Rodriguez et al., 2010, 2016). While miR396 also regulates other targets in groups of related species, only the miR396–*GRF* node is conserved in seed plants (Chorostecki et al., 2012). As a class of regulatory molecules, miR396 is up-regulated by various stress conditions, including drought, salinity, cold, and ultra-violet (UV)-B irradiation (Casadevall et al., 2013; Omidbakhshfard et al., 2015; Beltramino et al., 2018). It is possible that the increase in miR396 levels and the concomitant decrease in the level of *GRFs* mRNA are a part of the plant's strategy to reduce growth activities under various stresses, which are usually associated with decreased GA and thus increased levels of DELLAs.

Based on these clues, another study found that the negative response of miR396 to GA was a real cause of the positive response of *OsGRFs* to GA (Lu et al., 2020). There are 8 miR396 members in rice and 11 of 12 *OsGRFs* are miR396' targets. Under higher concentrations of GA in which SLR1 abundance is lower, the expression of OsmiR396 is also reduced, which in turn leads to higher levels of *OsGRFs* mRNA (Figure 1C). The positive correlation between the expression of miR396 and *SLR1* was further confirmed by detecting miR396 levels in the two key mutants, *gid1* (*gibberellin insensitive dwarf1*) and *eui* (*elongated uppermost internode*). GID1, a receptor of biologically active GAs, mediates the degradation of SLR1 under higher levels of active GAs. So, the level of SLR1 always keeps stable and can avoid being degraded in the *gid1* mutant (Ueguchi-Tanaka et al., 2005, 2008). EUI, a GA deactivating enzyme, helps to maintain higher levels of inactive GAs. So the level of SLR1 is reduced in the *eui* mutant due to higher levels of biologically active GAs (Zhu et al., 2006). OsmiR396' expression is higher in the *gid1* mutant and is lower in the *eui* mutant. The time course of gene expression also indicated that miR396 acts downstream of *SLR1* and upstream of GA-induced cell-cycle genes (Lu et al., 2020).

Using the promoter of OsmiR396a as bait, rice transcription factor INDETERMINATE DOMAIN2 (OsIDD2), was found to directly bind the promoter of OsmiR396a and can interact with SLR1 (Lu et al., 2020). As a family of transcription factors conserved among all land plants, indeterminate domains (IDDs) are defined by encoding four zinc-finger motifs utilized for binding DNA (Colasanti et al., 1998, 2006). IDDs have been reported to control diverse processes at various stages of plant development (Coelho et al., 2018). Overexpressing *OsIDD2* also led to a dwarf phenotype which is similar to transgenic lines overexpressing miR396 with a concomitant decrease in the level of *OsGRFs* mRNA (Huang et al., 2018; Lu et al., 2020). The interaction of OsIDD2 and SLR1 is indispensable for the expression of miR396 (Figure 1D) (Lu et al., 2020). A number of AtIDDs have been also shown to be capable of interacting with DELLA proteins *via* transactivation to regulate downstream genes in *Arabidopsis* (Feurtado et al., 2011; Fukazawa et al., 2014; Yoshida et al., 2014). So, SLR1 promoting miR396' expression with a subsequent decrease in the level of *OsGRFs* mRNA may provide more means for DELLA to decline GRFs' functions under stress. Because stress-induced DELLA, as the GA signaling hub, would be subjected to a large consumption due to extensive interactions between it and many other proteins. But DELLA may save its consumption by eliminating *GRFs* mRNA by promoting the expression of miR396 and thus curb the translation of GRFs which usually contain multiple members in plants.

## Conclusions and Future Perspective

Growing evidence has shown that *GRFs* play important roles in regulating the growth of a wide range of plant organs, usually through promoting the meristematic potential of primordial cells (Rodriguez et al., 2010, 2016). The positive regulatory roles of GRFs also depend on the partners of GIFs and SWI/SNF. However, plant growth and development are processes being subjected to exquisite regulation which relies on the balance of both negative and positive conditions. In the GRF-GIF-SWI/SNF module, GRFs are adjustable at both mRNA and protein levels (Figure 1). Hormones, specifically associated with the internal or external conditions, are usually at the upmost part of the signaling cascades in controlling genes' expression. DELLAs, which play pivotal roles in GA signaling and whose abundance is negatively regulated by GA, control the function of GRFs by means of both transactivation and sequestration. Through transactivation, DELLA can work with other factors (for example OsIDD2 in rice) to stimulate the expression of miR396 to finally decrease the abundance of *GRFs* mRNA (Figure 1D). For the pre-existing GRF proteins, DELLA can prevent them from interacting with GIF-SWI/SNF by sequestration (through a competitive manner).

It may still need more biochemistry works in the future to reveal full details of the interaction between DELLAs and GRFs, including what are key domains needed for the interaction and how DELLA and GIFs competitively interact with GRFs. Even though every component of this module is deeply conserved in “higher” plants, whether these are some nuances in the mechanism of this module working in other plant species still needs to be elucidated.

## Author Contributions

QL and YL conceived the review topics. YL wrote the manuscript. QL scrutinized the whole text. JZ made the figures. All authors contributed to the article and approved the submitted version.

## Funding

This work was supported by National Natural Science Foundation of China (No. 31271623) and the open fund of Key Laboratory of Plant Functional Genomics of the Ministry of Education (No. ML202004).

## Conflict of Interest

The authors declare that the research was conducted in the absence of any commercial or financial relationships that could be construed as a potential conflict of interest.

## Publisher's Note

All claims expressed in this article are solely those of the authors and do not necessarily represent those of their affiliated organizations, or those of the publisher, the editors and the reviewers. Any product that may be evaluated in this article, or claim that may be made by its manufacturer, is not guaranteed or endorsed by the publisher.
